# Clinicians in the Veterans Health Administration initiate gender-affirming hormone therapy in concordance with clinical guideline recommendations

**DOI:** 10.3389/fendo.2024.1086158

**Published:** 2024-05-10

**Authors:** Guneet K. Jasuja, Hill L. Wolfe, Joel I. Reisman, Varsha G. Vimalananda, Sowmya R. Rao, John R. Blosnich, Nicholas A. Livingston, Jillian C. Shipherd

**Affiliations:** ^1^ Center for Healthcare Organization & Implementation Research, Veteran Affairs (VA) Bedford Healthcare System, Bedford, MA, United States; ^2^ Section of General Internal Medicine, Boston University Chobanian & Avedisian School of Medicine, Boston, MA, United States; ^3^ Department of Health Law, Policy and Management, Boston University School of Public Health, Boston, MA, United States; ^4^ VA Center for Health Equity Research and Promotion, VA Pittsburgh Healthcare System, Pittsburgh, PA, United States; ^5^ Pain Research, Informatics, Multi-morbidities, and Education (PRIME) Center, VA Connecticut Healthcare System, West Haven, CT, United States; ^6^ Section of Biomedical Informatics and Data Science, Yale School of Medicine, New Haven, CT, United States; ^7^ Section of Endocrinology, Diabetes, Nutrition and Weight Management, Boston University Chobanian and Avedisian School of Medicine, Boston, MA, United States; ^8^ Department of Global Health, Boston University School of Public Health, Boston, MA, United States; ^9^ Suzanne Dworak-Peck School of Social Work, University of Southern California, Los Angeles, CA, United States; ^10^ National Center for PTSD, VA Boston Healthcare System, Boston, MA, United States; ^11^ Department of Psychiatry, Boston University Chobanian & Avedisian School of Medicine, Boston, MA, United States; ^12^ LGBTQ+ Health Program, Office of Patient Care Services, Department of Veterans Affairs, Washington, DC, United States

**Keywords:** gender-affirming hormone therapy, clinical guidelines, guideline concordance, transgender and gender diverse, veterans, Veterans Health Administration

## Abstract

**Background:**

Gender-affirming hormone therapy (GAHT) is a common medical intervention sought by transgender and gender diverse (TGD) individuals. Initiating GAHT in accordance with clinical guideline recommendations ensures delivery of high-quality care. However, no prior studies have examined how current GAHT initiation compares to recommended GAHT initiation.

**Objective:**

This study assessed guideline concordance around feminizing and masculinizing GAHT initiation in the Veterans Health Administration (VHA).

**Methods:**

The sample included 4,676 veterans with a gender identity disorder diagnosis who initiated feminizing (*n*=3,547) and masculinizing (*n*=1,129) GAHT between 2007 and 2018 in VHA. Demographics and health conditions on veterans receiving feminizing and masculinizing GAHT were assessed. Proportion of guideline concordant veterans on six VHA guidelines on feminizing and masculinizing GAHT initiation were determined.

**Results:**

Compared to veterans receiving masculinizing GAHT, a higher proportion of veterans receiving feminizing GAHT were older (≥60 years: 23.7% vs. 6.3%), White non-Hispanic (83.5% vs. 57.6%), and had a higher number of comorbidities (≥7: 14.0% vs. 10.6%). A higher proportion of veterans receiving masculinizing GAHT were Black non-Hispanic (21.5% vs. 3.5%), had posttraumatic stress disorder (43.0% vs. 33.9%) and positive military sexual trauma (33.5% vs.16.8%; all p-values<0.001) than veterans receiving feminizing GAHT. Among veterans who started feminizing GAHT with estrogen, 98.6% were guideline concordant due to no documentation of venous thromboembolism, or breast cancer. Among veterans who started spironolactone as part of feminizing GAHT, 98.1% were guideline concordant as they had no documentation of contraindication, including hyperkalemia or acute renal failure. Among veterans starting masculinizing GAHT, 90.1% were guideline concordant due to no documentation of contraindications, such as breast or prostate cancer. Hematocrit had been measured in 91.8% of veterans before initiating masculinizing GAHT, with 96.5% not having an elevated hematocrit (>50%) prior to starting masculinizing GAHT. Among veterans initiating feminizing and masculinizing GAHT, 91.2% had documentation of a gender identity disorder diagnosis prior to GAHT initiation.

**Conclusion:**

We observed high concordance between current GAHT initiation practices in VHA and guidelines, particularly for feminizing GAHT. Findings suggest that VHA clinicians are initiating feminizing GAHT in concordance with clinical guidelines. Future work should assess guideline concordance on monitoring and management of GAHT in VHA.

## 1 Introduction

Many transgender and gender diverse (TGD) individuals experience gender dysphoria, defined as a marked incongruence between one’s gender identity and sex assigned at birth, to the extent that the “disturbance causes clinically significant distress or impairment.” (1) Evidence-based treatments for alleviating gender dysphoria among TGD individuals include feminization or masculinization through interventions, such as gender-affirming hormone therapy (GAHT) and/or surgery, psychotherapy, and changes in gender identity or expression (2). Although not every TGD individual seeks gender-affirming treatments, among those who need or desire these medical treatments, GAHT is one of the most utilized gender-affirming medical interventions (3). Numerous studies show that GAHT can improve mental health and quality of life and lower the risk for suicidal ideation and suicide attempts among TGD individuals, including among TGD veterans (4–10).

Individual treatment plans should align with the unique transition-related goals of each TGD individual. GAHT is provided to induce feminizing or masculinizing changes to the body among individuals who desire these changes (11, 12). Estrogen is the primary hormone therapy for inducing feminization in TGD individuals (13, 14). However, estrogen alone is often not enough to achieve desirable androgen suppression, and adjunctive anti-androgenic therapy, such as spironolactone, is usually recommended by clinical guidelines (15, 16). Testosterone is the primary hormone therapy for inducing masculinization in TGD individuals (13, 17).

Though GAHT has a positive impact on mental health and quality of life in the TGD population (4–10), both feminizing and masculinizing GAHT have been shown to be associated with certain risks. Feminizing GAHT has been associated with increased risk for myocardial infarction, thrombogenic complications, and the potential risks of cancer and infertility (12, 18–21), while masculinizing GAHT has been associated with increased erythrocytosis and potential risks of cancer, hypertension and infertility (20, 22–24). Thus, GAHT is safe and effective when initiated and monitored in accordance with established clinical guidelines (25). As an example of a recommended guideline around masculinizing GAHT initiation, hematocrit levels need to be checked before initiation of testosterone (13, 15). This and similar aspects of care on GAHT initiation are measurable and improving the fidelity with which they are delivered may help improve population health for TGD individuals.

However, not much is known about how well clinicians follow guideline recommendations around initiation of GAHT among TGD individuals. Compared to the general population, TGD individuals are more likely to have served in the U.S. Armed Forces (3, 26, 27). Specifically, diagnoses related to transgender identity are five times more prevalent in the Veterans Health Administration (VHA) than in the general population (28). Thus, in accordance with VHA guideline recommendations, which could be assessed using structured data, we sought to assess guideline concordance around initiation of GAHT in VHA—one of the largest providers of medical care to the TGD population in the United States (29). Of note, VHA guidelines are unique to VHA and deviate from guidelines utilized by other organizations in several aspects (2, 15). For example, unlike non-VHA guidelines, VHA internal guidelines require that candidates for GAHT should:1) fulfill the diagnostic criteria for gender identity disorder, as determined by a qualified mental health care professional or other qualified professional with expertise in the treatment of transgender individual before initiation of GAHT and 2) have the capacity to make an informed consent (13). Understanding the concordance between current GAHT initiation prescribing practices in VHA and guideline recommendations is essential to determining the quality of GAHT care VHA is providing to its TGD population.

## 2 Methods

### 2.1 Data sources

We used outpatient and inpatient data for encounters between calendar year (CY) 2006 and 2018 from the VHA’s Corporate Data Warehouse (CDW) for demographics, and medical diagnoses, medical history, laboratory results, and outpatient prescriptions. This study was approved by the VA Bedford Healthcare System Institutional Review Board.

### 2.2 Study population

**Figure 1:**
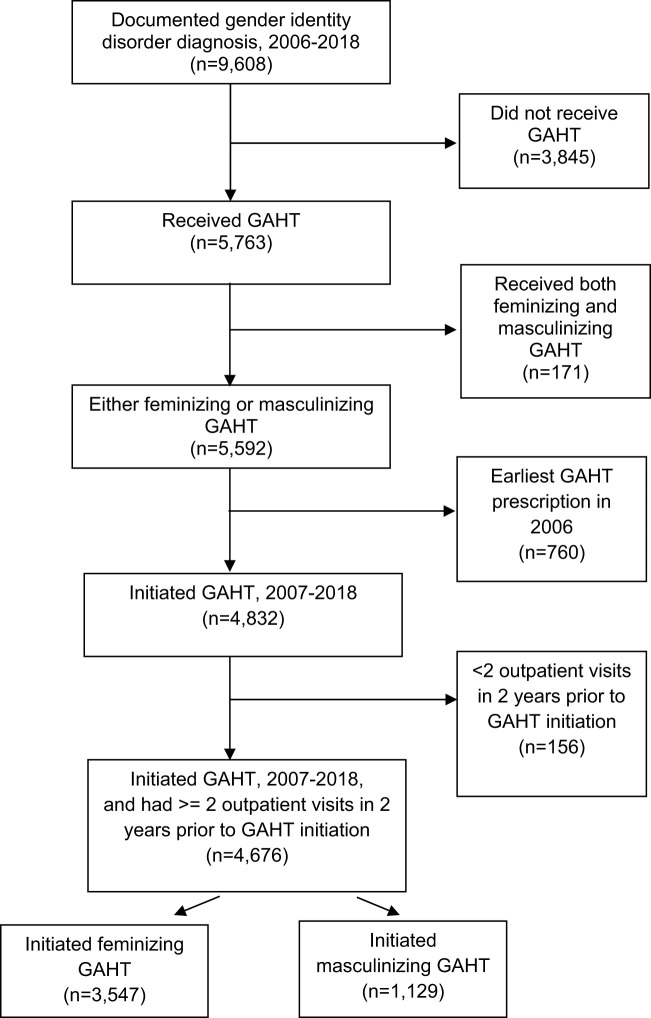
Study population. GAHT, gender-affirming hormone therapy.

Our study population included veterans with a gender identity disorder diagnosis (**Supplementary Table 1**) documented in the VHA and who received a feminizing or masculinizing GAHT prescription in VHA between CY 2006 and 2018. Of the 9,608 veterans with a documented gender identity disorder diagnosis between 2006 and 2018, 4,359 veterans had a feminizing GAHT prescription and 1,233 had a masculinizing GAHT prescription. An additional 171 veterans who received both feminizing and masculinizing GAHT were excluded. Of these 171 veterans, 162 veterans did not receive these therapies simultaneously. Switching between these therapies for these small number of veterans (~1.7% of our cohort with gender identity disorder diagnoses) could occur for a variety of reasons, including identifying as non-binary, genderqueer, or another related identity (30). Since in this study we examined guideline concordance on GAHT initiation, we looked back to the beginning of the study period i.e., to 2006 to ensure that the feminizing or masculinizing GAHT prescription each individual received between 2006-18 was their first GAHT prescription in VHA. We excluded 760 veterans who received their earliest GAHT prescription in the first year of the study period (2006) as we could not look back further to check if the GAHT prescription they received in 2006 was their earliest GAHT prescription in VHA. Details of the hormone therapy regimen (masculinizing and feminizing), including dosage levels have been reported previously (31). To establish a cohort of VHA users, we further required veterans who received a GAHT prescription (either feminizing or masculinizing) between 2007-2018 to have at least two outpatient visits in the two years prior to the earliest GAHT prescription date. After applying this outpatient visit exclusion (n=156), our sample consisted of a total of 4,676 veterans. Of these 4,676 veterans, 3,547 veterans initiated feminizing GAHT and 1,129 initiated masculinizing GAHT between 2007-18 (see **Figure 1** for the step-by-step representation of the study population).

### 2.3 Socio-demographics

We examined the distribution of several socio-demographics with feminizing and masculinizing GAHT in the VHA, including age, marital status, race/ethnicity, urban/rural residence, type of facility where the veteran received care (community-based outpatient clinic (CBOC) or VHA hospital), and poverty of zip code of residence, and region of site (Midwest, Northeast, South, West) at the time of the earliest GAHT prescription. The poverty of zip code of residence was defined as the percentage of households in the corresponding census zip code having income below 100% of the poverty level as reported in the 2015 American Community Survey (32). Type of facility was determined from all outpatient visits in the two years prior to the veteran’s earliest GAHT prescription.

### 2.4 Comorbidities

We evaluated the distribution of physical and mental health conditions with each type of GAHT from the date of the individual’s earliest GAHT prescription until the end of the study period. A condition was considered present if the individual had at least two instances of a diagnosis separated by seven days or more (33). The presence of these conditions was based on inpatient and outpatient diagnoses using International Classification of Diseases (ICD)-9 and ICD-10 codes as detailed in **Supplementary Table 2**. In addition to individual conditions that may impact hormone prescriptions, we also used the Elixhauser Comorbidity Index, a composite score of 31 comorbidities, as a comorbidity count variable (34).

### 2.5 Social stressors

Documented social stressors, obtained from three structured data sources within the CDW (35), were assessed from the date of the gender identity disorder diagnosis until the end of the study period. First, we examined ICD-9 and ICD-10 codes related to social stressors (**Supplementary Table 3A**). Second, we identified VHA codes indicating receipt of services (e.g., VHA Homeless Programs) (**Supplementary Table 3B**) related to social stressors. Third, we examined progress notes authored by healthcare staff which included presenting issues (e.g., being unemployed) and other stressors (e.g., lack of social support) commonly inquired about during referrals (**Supplementary Table 3C**). Veterans were coded as having a documented social stressor if they had any indicators from any of the three structured data sources. We coded seven categories of documented social stressors: experience of violence, social/familial problems, housing instability, employment/financial stressors, legal issues, nonspecific psychosocial needs, and lack of access to care/transportation reported by TGD individuals in prior studies (36–38).

### 2.6 Characteristics of GAHT regimen

Characteristics of the masculinizing and feminizing GAHT regimen were determined for the GAHT that was prescribed on the date of initiation. Specifically, regimens were classified by type of hormone (estrogen, estradiol, spironolactone, and progesterone for feminizing GAHT and testosterone for masculinizing GAHT) and route of administration (oral, topical/transdermal, injection). Duration of GAHT was calculated as the time between GAHT initiation and the latest GAHT prescription fill date.

### 2.7 Ascertainment of guideline concordance around GAHT initiation in VHA

To compare GAHT initiation in VHA to what is recommended by guidelines, we primarily focused on six guidelines recommended by internal VHA clinical recommendations per the VHA Pharmacy Benefits Management Services, which could be measured using VHA structured data. (**Supplementary Table 4**) (13).

Using CDW data, we calculated the proportion of guideline concordant veterans for each of the six VHA internal clinical guidelines separately for initiating feminizing and masculinizing GAHT. Concordance for each of these six guidelines was calculated by dividing the number of veterans whose care aligned with the specific guideline (numerator) by the eligible veterans who received either feminizing or masculinizing GAHT per the specific guideline (denominator).

For assessing contradindications associated with estrogen and testosterone therapy, we used a look-back period of two years starting from the earliest feminizing or masculinizing GAHT prescription between 2007-2018. For assessment of pregnancy before initiating testosterone, we used a nine month look-back period. For assessing contraindications, including hyperkalemia and acute renal failure associated with spironolactone, we used a two month look-back period starting from the earliest feminizing prescription involving spironolactone between 2007-2018. We used a two month look-back period for hyperkalemia and acute renal failure since these conditions are generally acute and treated to resolution. Thus, these contraindications are relevant only for being present at the time of prescribing of spironolactone. For assessing documentation of hematocrit and hematocrit levels before start of testosterone, we used a look-back period of one year starting from the earliest testosterone prescription between 2007-2018.

### 2.8 Statistical analyses

Descriptive statistics for the veteran characteristics and characteristics of the GAHT regimen were generated for the study population stratified by the type of GAHT. Comparisons between feminizing and masculinizing GAHT on descriptive or GAHT regimen characteristics were assessed by a two-sided chi-squared test. Analyses were conducted using SAS, version 9.4 (SAS Institute Inc., Cary, NC).

## 3 Results

### 3.1 Descriptive characteristics by type of GAHT

As reported in **Table 1**, a higher percentage of veterans receiving feminizing GAHT were older (20-29 years: 16.4% vs. 41.7%; 30-39 years: 20.0% vs. 28.1%; 40-49 years: 16.1% vs. 12.5%; 50-59 years: 23.8% vs. 11.4%; ≥60 years: 23.7% vs. 6.3%) and White non-Hispanic (83.5% vs. 57.6%) as compared to those receiving masculinizing GAHT. Further, a higher percentage of veterans on feminizing GAHT had a documented diagnosis of atherosclerosis (6.4% vs. 2.4%), diabetes (17.5% vs. 9.2%), hyperlipidemia (41.3% vs. 26.0%), hypertension (35.0% vs. 17.9%), and tobacco use (28.0% vs. 21.2%; all p-values <0.001) compared to veterans using masculinizing GAHT. Veterans using feminizing GAHT tended to have a higher Elixhauser Comorbidity Index (0,1: 31.3% vs. 40.2%; 2,3: 31.1% vs. 28.7%; 4-6: 23.6% vs. 20.5%; ≥7: 14.0% vs. 10.6%), while feminizing and masculinizing GAHT groups were comparable on social stressors despite the age difference. Conversely, a higher percentage of veterans receiving masculinizing GAHT were Black non-Hispanic (21.5% vs. 3.5%), had a documented diagnosis of posttraumatic stress disorder (43.0% vs. 33.9%), and had a positive screening for military sexual trauma (33.5% vs.16.8%; all p-values<0.001) compared to veterans receiving feminizing GAHT.

**Table 1:** Descriptive characteristics by type of gender-affirming hormone therapy (GAHT).

Variable	Feminizing GAHT N=3,547	Masculinizing GAHT N=1,129	p-value^a^
	n (%)		
Age			<0.001
21-29	580 (16.4%)	471 (41.7%)	
30-39	710 (20.0%)	317 (28.1%)	
40-49	571 (16.1%)	141 (12.5%)	
50-59	845 (23.8%)	129 (11.4%)	
≥60	841 (23.7%)	71 (6.3%)	
Race/Ethnicity			<0.001
Asian	48 (1.4%)	28 (2.5%)	
Black, non-Hispanic	124 (3.5%)	243 (21.5%)	
Hispanic	131 (3.7%)	91 (8.1%)	
White, non-Hispanic	2,963 (83.5%)	650 (57.6%)	
Other/Unknown	281 (7.9%)	117 (10.4%)	
Location of residence			0.025
Rural	860 (24.2%)	237 (21.0%)	
Urban	2,687 (75.8%)	892 (79.0%)	
Region			<0.001
Midwest	762 (21.5%)	184 (16.3%)	
Northeast	385 (10.9%)	119 (10.5%)	
South	1,090 (30.7%)	424 (37.6%)	
West	1,310 (36.9%)	402 (35.6%)	
% Poverty in Zip Code of Residence			0.002
0-5.5%	861 (24.3%)	221 (19.6%)	
5.5-9.2%	907 (25.6%)	276 (24.4%)	
9.2-14.1%	942 (26.6%)	320 (28.3%)	
14.1%+	837 (23.6%)	312 (27.6%)	
Marital status			0.117
Married	941 (26.5%)	273 (24.2%)	
Not Married	2,606 (73.5%)	856 (75.8%)	
Location of Care			0.204
All care received at CBOC	55 (1.6%)	12 (1.1%)	
All care received at VHA hospital	530 (14.9%)	158 (14.0%)	
Care received at CBOC & VHA hospital	2,962 (83.5%)	959 (84.9%)	
Health Conditions			
Alcohol Use Disorder	452 (12.7%)	160 (14.2%)	0.215
Atherosclerosis	226 (6.4%)	27 (2.4%)	<0.001
Depression	2,328 (65.6%)	706 (62.5%)	0.058
Diabetes	622 (17.5%)	104 (9.2%)	<0.001
Drug Use Disorder	540 (15.2%)	179 (15.9%)	0.609
Human Immunodeficiency Virus	76 (2.1%)	10 (0.9%)	0.006
Hyperlipidemia	1,465 (41.3%)	294 (26.0%)	<0.001
Hypertension	1,243 (35.0%)	202 (17.9%)	<0.001
Myocardial Infarction	60 (1.7%)	7 (0.6%)	0.008
Posttraumatic Stress Disorder	1,204 (33.9%)	485 (43.0%)	<0.001
Tobacco Use	994 (28.0%)	239 (21.2%)	<0.001
Venous Thromboembolism	77 (2.2%)	16 (1.4%)	0.114
Elixhauser Comorbidity Index			<0.001
0, 1	1,110 (31.3%)	454 (40.2%)	
2, 3	1,104 (31.1%)	324 (28.7%)	
4-6	836 (23.6%)	231 (20.5%)	
≥7	497 (14.0%)	120 (10.6%)	
Social Stressors			
Violence	29 (0.8%)	14 (1.2%)	0.195
Social/Familial Problems	215 (6.1%)	79 (7.0%)	0.259
Housing Instability	324 (9.1%)	87 (7.7%)	0.140
Employment/Financial Stressors	319 (9.0%)	100 (8.9%)	0.889
Legal Issues	63 (1.8%)	17 (1.5%)	0.542
Nonspecific Psychosocial Needs	317 (8.9%)	115 (10.2%)	0.207
Lack of Access to Care/Transportation	49 (1.4%)	10 (0.9%)	0.194
Count of Social Stressors			0.005
0	2,920 (82.3%)	898 (79.5%)	
1	261 (7.4%)	120 (10.6%)	
2	173 (4.9%)	58 (5.1%)	
≥3	193 (5.4%)	53 (4.7%)	
Military Sexual Trauma			<0.001
Positive screening	597 (16.8%)	378 (33.5%)	
Negative screening	2,950 (83.2%)	751 (66.5%)	
Suicide Attempt			0.157
Documented	439 (12.4%)	122 (10.8%)	
Not documented	3,108 (87.6%)	1,007 (89.2%)	
Mental Health Visits^b^			
On or before initiation	2,948 (83.1%)	941 (83.3%)	0.854
During GAHT	2,857 (80.5%)	926 (82.0%)	0.273
None in either period	114 (3.2%)	32 (2.8%)	0.523

a p-value, from two-sided chi-squared test; ^b^outpatient visits in 1-year period prior to GAHT initiation.

CBOC, Community-based outpatient clinic.

### 3.2 Type, route of administration and duration of GAHT

Of the 3,547 veterans who received feminizing GAHT, 55.5% (n=1,967) veterans were prescribed either estradiol or estrogen at GAHT initiation (**Table 2**). Of these 1,967 veterans, 67% (n=1,320) had spironolactone added between 2007-2018. Approximately 33% of these veterans (n=647) did not receive spironolactone in the study period. Further, approximately 12% (n=417) of veterans on feminizing GAHT received only spironolactone at the time of GAHT initiation. Of these 417, 97.6% (n=407) also received estrogen or estradiol between 2007-18 while 2.4% (n=10) did not receive estrogen or estradiol. Thus, 35.3% of individuals (32.9% did not receive spironolactone and 2.4% did not receive estrogen/estradiol) did not receive both estrogen and spironolactone (combination therapy) when initiating feminizing GAHT. In data not shown, approximately 26% of those on feminizing GAHT received progesterone. Approximately 99% of the feminizing prescriptions were estrogen or estradiol and 1% were ethinyl estradiol. There were no prescriptions for ciproterone, which is the most commonly used antiandrogen in Europe.

**Table 2:** Type, route of administration, and duration of gender-affirming hormone therapy (GAHT) at GAHT initiation.

Variable	Feminizing GAHT N=3,547	Masculinizing GAHT N=1,129
	n (%)	n (%)
Type of GAHT		
Feminizing GAHT		
Estrogen or estradiol only	1,967 (55.5%)	–
Added spironolactone ^a^	1,320 (67.1%)	
Did not add spironolactone	647 (32.9%)	–
Estrogen/estradiol and spironolactone	1,163 (32.8%)	–
Spironolactone	417 (11.8%)	–
Added estrogen/estradiol ^b^	407 (97.6%)	
Did not add estrogen/estradiol	10 (2.4%)	–
Breast cancer screening ^c^	1,194 (29.8%)	–
Bone densitometry ^d^	766 (21.8%)	–
Masculinizing GAHT		
Testosterone, Any^e^	–	1,129 (100.0%)
Testosterone enanthate	–	27 (2.4%)
Testosterone cypionate	–	993 (88.0%)
Testosterone undecanoate	–	0 (0%)
Testosterone, unspecified	–	109 (9.7%)
Testosterone gel	–	4 (0.4%)
Testosterone patch	–	99 (8.8%)
Prostate specific antigen levels^f^	–	207 (18.3%)
Breast cancer screening^c^	–	228 (9.2%)
Bone densitometry ^d^	–	50 (12.0%)
Route of adminstration^g^ and Average daily dosage		
Oral (capsules or tablets)	3,113 (87.8%)	–
Estrogen or estradiol Estrogen/estradiol and spironolactone	1.7 mg 76.7 mg	–
Spironolactone only	79.5 mg	–
Topical/transdermal (gels and patches)	1,732 (48.8%)	169 (15.0%)
Estradiol	0.06 mg	–
Testosterone	–	4.4 mg
Injection (intramuscular or subcutaneous)	829 (23.4%)	1,072 (95.0%)
Estrogen or estradiol	17.8 mg/mL	–
Testosterone	–	224.4 mg/mL
Duration of GAHT		
<1 yr.	322 (9.1%)	117 (10.4%)
1-2 yr.	225 (6.3%)	58 (5.1%)
2-3 yr.	534 (15.1%)	174 (15.4%)
3-4 yr.	494 (13.9%)	182 (16.1%)
4+ yr.	1,972 (55.6%)	598 (53.0%)
Laboratory Values^h^		
Creatinine (mg/dL)	0.97 (0.59)	0.96 (0.19)
ALT (U/L)	26.8 (19.4)	26.3 (17.6)
AST (U/L)	22.4 (12.6)	24.7 (17.2)
Hematocrit (%)	41.7 (3.7)	44.7 (4.0)
LDL (mg/dL)	98.2 (37.9)	102.1 (39.5)
HDL (mg/dL)	49.4 (15.3)	44.3 (12.8)
Prolactin (ng/mL)	12.0 (8.9)	n/a

a After initiation, added spironolactone in the study period (2007-2018).

b After initiation, added estrogen/estradiol in the study period (2007-2018).

c Patients aged 50-65 years within 2 years of GAHT initiation.

d Patients age>=65 years and on GAHT for at least 1.5 years.

e Patients could have multiple prescriptions of different types of testosterone.

f At least 1 laboratory test for prostate specific antigen within 2 years after GAHT initiation.

g Multiple prescriptions for a patient are possible.

h Average (standard deviation) of latest value in the year post GAHT initiation for each patient.

ALT, alanine aminotransferase.

AST, aspartate aminotransferase.

LDL, low density lipids.

HDL, high density lipids.

Compared to approximately 30% of veterans on feminizing GAHT aged 50-65 years who had documentation of breast cancer screening after GAHT initiation, only 9.2% on masculinizing GAHT had documentation of this screening after initiation. Also, compared to approximately 22% of veterans on feminizing GAHT aged 65 years or older who had documentation of bone densitometry after GAHT initiation, only 12% on masculinizing GAHT had this documentation after initiation. Further, only 18% of veterans on masculinizing GAHT had their prostate specific antigen levels monitored after GAHT initiation. In terms of route of administration, oral capsules or tablets were the predominant route of administration for those using feminizing GAHT (87.8%), while injection was the predominant route of administration for those using masculinizing GAHT (95%). Further, in most TGD veterans (55.6% using feminizing GAHT and 53% using masculinizing GAHT) in the cohort report a consistent and persistent use of GAHT for a period superior to 4 years.

### 3.3 Guideline concordance on GAHT initiation

As reported in **Table 3**, in accordance with VHA guidelines, among veterans who started feminizing GAHT with estrogen, 98.6% were guideline concordant in terms of not having a documentation of venous thromboembolism or, breast cancer, at GAHT initiation. Further, among veterans who started spironolactone, 98.1% were guideline concordant with not having a documentation of hyperkalemia or acute renal failure at GAHT initiation. Among veterans initiating testosterone, 90.4% did not have documentation of contraindications, such as breast or prostate cancer at initiation. Approximately 10% of veterans (n=108) on masculinizing GAHT had documentation of a contraindication. Of these 10% veterans, while 19.4% (n=21) had breast cancer, none had prostate cancer.

**Table 3:** Guideline concordance on gender-affirming hormone therapy (GAHT) initiation.

Type of GAHT	Guidelines	Numerator N	Denominator N	Guideline concordance %
VHA Guidelines				
Feminizing	Do not start estrogen if patient has history of venous thromboembolism* or, breast cancer**	3,499	3,441	98.6%
Feminizing	Do not start spironolactone if patient has a history of hyperkalemia** or acute renal failure**	2,312	2,356	98.1%
Feminizing	Documentation of a gender identity disorder diagnosis required prior to starting feminizing GAHT	3,284	3,547	92.6%
Masculinizing	Do not start testosterone if patient is pregnant* or has history of breast cancer**, prostate cancer**, severe renal or hepatic disease**, or myocardial infraction**, stroke**, or heart failure**	1,021	1,129	90.4%
Masculinizing	Documentation of a gender identity disorder diagnosis required prior to starting masculinizing GAHT	982	1,129	87.0%
Masculinizing	Check hematocrit before starting testosterone	1,036	1,129	91.8%
Masculinizing	Do not start testosterone if patient has uncontrolled or untreated erythrocytosis*	1,000	1,036	96.5%
Feminizing and Masculinizing	Documentation of a gender identity disorder diagnosis required prior to starting either feminizing or masculinizing GAHT	4,266	4,676	91.2%

Numerator, Patients whose care aligned with the specific guideline; Denominator, Eligible patients who received either feminizing or masculining GAHT as per the specific guideline; Denominator for feminizing VHA guideline with spirinolactone is limited to patients who intiated spironolactone; Uncontrolled or untreated erythrocytosis: hematocrit > 50% (15); Denominator for masculinizing guideline on hematocrit >50% (15) is limited to patients who had their hemotocrit checked (n=1,036). *absolute contraindication as per non-VHA guidelines; (2, 15) **relative contraindication as per non-VHA guidelines (2, 15).

As recommended by VHA guidelines, approximately 91% of the veterans had documentation of a gender identity disorder diagnosis prior to initiating either feminizing (92.6%) or masculinizing (87.0%) GAHT. While 7.4% of veterans who initiated feminizing GAHT and 13.0% of individuals who initiated masculinizing GAHT did not have a documentation of a gender identity disorder diagnosis before initiating GAHT, however, all these veterans had a gender identity disorder diagnosis documented at some time in study period after receipt of their first GAHT prescription, with a median interval of 154 days for feminizing and 715 days for masculinizing GAHT. Further, hematocrit, which is recommended to be measured before initiating testosterone, was measured in approximately 92% of veterans, while 96.5% of these veterans did not have an elevated hematocrit (>50%) prior to starting testosterone.

### 3.4 Longitudinal trends in the number of veterans on feminizing and masculinizing GAHT

**Figure 2:**
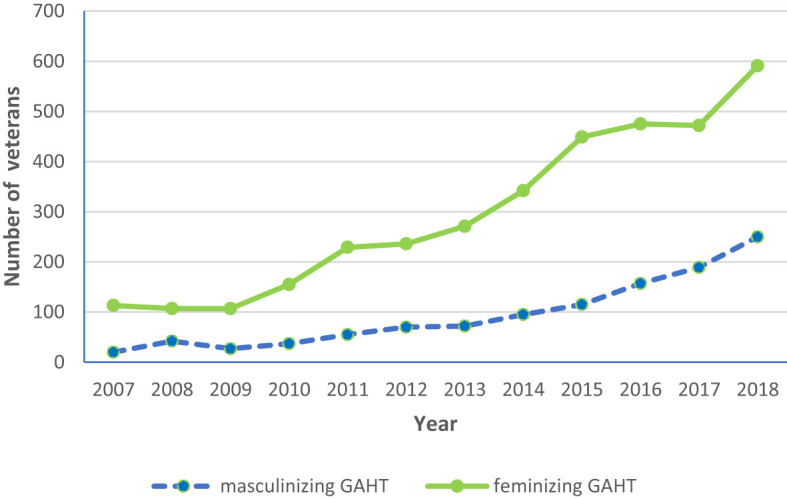
Longitudinal trends in the number of veterans on feminizing and masculinizing gender-affirming hormone therapy (GAHT) (2007–18).

As shown in **Figure 2**, the number of veterans on feminizing GAHT increased from 113 in 2007 to 591 in 2018. Although there was a predominance of veterans on feminizing GAHT throughout this period, the number of veterans on masculinizing GAHT also increased from 20 in 2007 to 250 in 2018.

## 4 Discussion

Using national VHA data, we observed high concordance between current GAHT initiation practices in VHA and recommended VHA and non-VHA guidelines on initiation GAHT. For veterans receiving feminizing GAHT, a low proportion of veterans had documented contraindications (3.0% with estrogen; 1.9% with spironolactone) or lack of required documentation of gender identity disorder diagnosis (7.4%) when initiating feminizing GAHT. There was lower guideline concordance around initiation of masculinizing GAHT. Approximately 10% of veterans had documentation of a contraindication (history of breast cancer, prostate cancer, etc.), 13% of veterans lacked documentation of a gender identity disorder diagnosis, and 8.2% did not have hematocrit levels checked at masculinizing GAHT initiation. Further, of these 8.2% veterans, 3.5% had hematocrit levels above 50% before starting masculinizing GAHT. Of note, a low percentage of veterans had documentation of breast cancer screening (~30%) and bone densitometry (~22%) after initiation of feminizing GAHT and an even lower percentage had documentation of this screening (9.2%), densitometry (12%), and prostate specific antigen levels (18%) after initiation of masculinizing GAHT. Thus, findings from our study suggest that VHA clinicians are effectively following guideline recommendations around GAHT initiation, particularly with feminizing therapies. To our knowledge, this is first study to use large, nationally representative data to measure how well clinicians follow key guidelines pertaining to GAHT initiation.

Our finding of lower guideline concordance around masculinizing GAHT initiation compared to feminizing GAHT initiation suggests that VHA needs to strive for improvement in assessing contraindications, hematocrit and documenting gender identity disorder diagnoses before starting masculinizing GAHT. In particular, since masculinizing GAHT is expected to stimulate erythrocytosis due to significant increases in hematocrit following the initiation of masculinizing GAHT (39), clinical guidelines recommend checking of hematocrit levels before initiating masculinizing GAHT (13, 15). Conditions, such as obstructive sleep apnea or tobacco use should be excluded as secondary reasons to elevated hematocrit before and during masculinizing GAHT and medical teams must be trained on identification of elevated hematocrit. We speculate that these findings of lower guideline concordance around masculinizing GAHT initiation may be because VHA clinicians are more familiar and knowledgeable with clinical guidelines on feminizing GAHT initiation considering that majority of the veteran population are assigned a male sex at birth (40, 41). Hence, we found that 3 times as many TGD veterans initiated feminizing GAHT as initiated masculinizing GAHT. In contrast to this finding in our study, a significant increase in number of transgender men compared to transgender women in recent years has been noted in the literature (42).

Our results support that feminizing GAHT initiation is primarily conducted in a manner concordant with practice guidelines. However, findings from our study also suggest that 35.3% of individuals (32.9% did not receive spironolactone and 2.4% did not receive estrogen/estradiol) did not receive both estrogen and spironolactone (combination therapy) in VHA during the entire study period when initiating feminizing GAHT. Use of this combination therapy is recommended by clinical guidelines (15). In a previous study, we found that the combination therapy using estrogen and spironolactone became more common in VHA over time, with the proportion of veterans receiving this therapy increasing from 39% to 69% from 2006 to 2017 (43). Thus, it is likely that many clinicians are still bringing their practice patterns in line with guideline recommendations on use of combination therapy for feminizing GAHT. Combination therapy can help block the effects of testosterone and achieve more effective feminization (44), however, many individuals may not need or want to take spironolactone. Specifically, some individuals may have undergone surgical orchiectomy or are using GnRH analogs, both of which are very effective at blocking the effects of testosterone. In our study, 14% of veterans on feminizing GAHT switched to GnRH analogs from spironolactone after GAHT initiation. Hence, spironolactone might not be needed for these individuals (2, 15). Other individuals may have kidney failure that precludes use of spironolactone (45). Additionally, individuals with a non-binary gender identity may not have a goal of maximizing feminine characteristics (46), and therefore may not need spironolactone to achieve their treatment goals.

Clinical practice guidelines exist to help clinicians identify appropriate candidates for GAHT and can act as a framework for choosing treatment regimens and managing health during initiation of these medications. However, these guidelines are designed to provide general guidance and are not intended to dictate the treatment of an individual. Treatment decisions should consider each individual’s circumstances and deviations from guidelines may be clinically indicated in specific individuals. Thus, guidelines, such as the World Professional Association for Transgender Health allow for some flexibility based on unique risk benefit analyses of each individual (2). VHA has potentially less flexible guidelines with respect to past co-morbidities that are not followed at other organizations. For example, a myocardial infarction 15 years ago with no recurrences would not completely rule out treatment with testosterone. It is likely that many veterans who should be getting GAHT are being denied access to this therapy based on less flexibility of the VHA guidelines.

Participation of clinicians in TGD care training programs conducted by VHA may contribute to a greater likelihood of following practice guidelines and conducting necessary screenings around GAHT initiation in VHA. Since VHA began covering gender-affirming related care in 2011 (46), VHA has made several system-level changes in advancing clinical competence around in TGD health (47). These changes in the VHA have included creation of an internal website where VHA clinicians can access information on TGD healthcare, such as links to policies and professional society guidelines. Twelve online trainings in various aspects of transgender health are also available on demand (48). VHA also provides national electronic consults (e-consults), a clinical consultation program that allows any VHA clinician to connect with an interdisciplinary team of clinicians through the electronic medical record regarding patient-specific clinical advice (49). Further, to provide more intensive training to clinicians, a national Specialty Care Access Network-Extension of Community Healthcare Outcomes (SCAN-ECHO) training program has been used to train interdisciplinary teams of clinicians in the care of TGD veterans throughout the VHA system (50, 51). Of note, VHA has achieved such widespread access to GAHT by purposely using the training programs mentioned to build capacity in the non-endocrinology clinician pool, enabling and empowering psychiatrist, primary care clinicians, internists, etc. to be the primary providers of GAHT.

Although clinical guidelines for the care of TGD individuals exist, quality measures to evaluate the care specific to this population are lacking (52, 53). Guideline recommendations used in this study can inform the development of valid and reliable quality measures around GAHT initiation. Quality measures are tools that help measure healthcare processes, outcome, patient perceptions, and other factors with the goal of delivering effective, safe, efficient, patient-centered, equitable, and timely care (54). Implementing these quality measures can serve as a basis for improving GAHT care (55) and ultimately reducing inequities in the TGD population in healthcare systems, including in VHA.

In addition to the higher proportion of guideline concordance on feminizing GAHT initiation, in our study, we also found that a higher proportion of veterans receiving feminizing GAHT were older, White non-Hispanic, and had a higher number of co-morbidities compared to veterans receiving masculinizing GAHT. This finding is likely indicative of the VHA’s high proportion of veterans who are older, White, and assigned male at birth (40, 41, 56). Further, similar to our study, transfeminine people and transgender veterans were reported to have a higher likelihood of most chronic conditions, including atherosclerosis, diabetes, HIV, hypertension, myocardial infarction, and tobacco use (57, 58). Meanwhile, our study also revealed a higher proportion of veterans receiving masculinizing GAHT were Black non-Hispanic, had documentation posttraumatic stress disorder, and screened positive for military sexual trauma. Racial disparities in mental and physical comorbidities in Black transgender veterans compared to White transgender veterans have been noted previously (59). Our finding of a higher proportion of veterans receiving masculinizing GAHT having posttraumatic stress disorder and military sexual trauma aligns with prior studies in which transmasculine people had higher likelihood of posttraumatic stress disorder (57) and transmasculine veterans had higher rates of military sexual trauma (60, 61) as compared to transfeminine people and transfeminine veterans, respectively.

While this study is an important first step in taking an inventory of GAHT care received by TGD veterans in VHA, our study has several limitations. Our identification of TGD veterans primarily relied on transgender-related diagnosis codes rather than patient’s self-reported gender identity. This has likely biased our sample toward patients who are engaged in care and treatment. However, in a prior study, we confirmed through chart review validation that veterans identified through these diagnoses were appropriately categorized as TGD veterans (31). Second, we did not capture GAHT prescriptions or care in VHA community care (care paid by VHA in the community) or covered by non-VHA payers (e.g., private insurance). Third, we realize that not all aspects of GAHT care are amenable to quality measures using automated data, such as whether patient is fully informed of risks and benefits of GAHT (13). Such aspects of quality of care, although important, would require chart review to assess, and are a topic for a future study. Here, we focused on GAHT initiation measures that could be easily operationalized in structured data. Fourth, our study did not assess patients who are interested in GAHT but denied this care. Future work should assess if the refusal of GAHT is occurring based on recommended guidelines or for other reasons. Fifth, since VHA does not currently include coverage for gender-affirming surgery in the medical benefits package, we cannot reliably ascertain gender-affirming surgery history in the data (46). Therefore, we cannot examine whether clinicians are following recommendations on anti-androgenic therapy suspension after these surgeries or if route or dose of estrogen administration was modified potentially due to these surgeries. Sixth, we used gender identity disorder diagnoses to identify transgender and gender diverse veterans in our study cohort from VHA structured data, Hence, it is not possible to identify the specific gender identity of veterans in our cohort. Finally, VHA structured data does not include fertility preservation data. Hence, it cannot be examined if fertility preservation is a fragility point during follow-up for TGD individuals after GAHT initiation.

In conclusion, we observed high concordance between initiation prescribing practices in VHA and clinical guideline recommendations for GAHT, particularly for feminizing therapy. Understanding the quality of care received at GAHT initiation among TGD individuals is important to prevent adverse health outcomes at the point of initiation in this population. Future research should examine predictors of guideline discordance and health outcomes associated with guideline discordance in TGD veterans initiating feminizing and masculinizing GAHT. Further, guideline concordance around GAHT monitoring and management in VHA and other healthcare systems should also be examined.

## Data availability statement

A deidentified data set supporting the conclusions of this article will be made available by the authors, without undue reservation.

## Author contributions

GJ was involved in the study conceptualization, writing, supervision and funding acquisition. GJ and HW were involved in methodology. JR analyzed the data. HW, VV, SR, JB, NL, and JS critically reviewed and approved the final manuscript. All authors contributed to the article and approved the submitted version.
