# Corrigendum: Clinicians in the Veterans Health Administration initiate gender-affirming hormone therapy in concordance with clinical guideline recommendations

**DOI:** 10.3389/fendo.2024.1448887

**Published:** 2024-07-08

**Authors:** Guneet K. Jasuja, Hill L. Wolfe, Joel I. Reisman, Varsha G. Vimalananda, Sowmya R. Rao, John R. Blosnich, Nicholas A. Livingston, Jillian C. Shipherd

**Affiliations:** ^1^ Center for Healthcare Organization & Implementation Research, Veteran Affairs (VA) Bedford Healthcare System, Bedford, MA, United States; ^2^ Section of General Internal Medicine, Boston University Chobanian & Avedisian School of Medicine, Boston, MA, United States; ^3^ Department of Health Law, Policy and Management, Boston University School of Public Health, Boston, MA, United States; ^4^ VA Center for Health Equity Research and Promotion, VA Pittsburgh Healthcare System, Pittsburgh, PA, United States; ^5^ Pain Research, Informatics, Multi-morbidities, and Education (PRIME) Center, VA Connecticut Healthcare System, West Haven, CT, United States; ^6^ Section of Biomedical Informatics and Data Science, Yale School of Medicine, New Haven, CT, United States; ^7^ Section of Endocrinology, Diabetes, Nutrition and Weight Management, Boston University Chobanian and Avedisian School of Medicine, Boston, MA, United States; ^8^ Department of Global Health, Boston University School of Public Health, Boston, MA, United States; ^9^ Suzanne Dworak-Peck School of Social Work, University of Southern California, Los Angeles, CA, United States; ^10^ National Center for PTSD, VA Boston Healthcare System, Boston, MA, United States; ^11^ Department of Psychiatry, Boston University Chobanian & Avedisian School of Medicine, Boston, MA, United States; ^12^ LGBTQ+ Health Program, Office of Patient Care Services, Department of Veterans Affairs, Washington, DC, United States

**Keywords:** gender-affirming hormone therapy, clinical guidelines, guideline concordance, transgender and gender diverse, veterans, Veterans Health Administration

In the published article, there was an error. A sentence in the results sub-section of the abstract did not match with a sentence in the results section 3.3 “Guideline concordance on GAHT initiation” on page 5 of the published article.

A correction has been made to the **Results** section in the **Abstract**. This sentence previously stated: “Among veterans who started feminizing GAHT with estrogen, 97.0% were guideline concordant due to no documentation of venous thromboembolism, breast cancer, stroke, or myocardial infarction.”

The corrected sentence appears below.

“Among veterans who started feminizing GAHT with estrogen, 98.6% were guideline concordant due to no documentation of venous thromboembolism, or breast cancer.”

The authors apologize for this error and state that this does not change the scientific conclusions of the article in any way. The original article has been updated.

