# Supplementary material for: Clinicians in the Veterans Health Administration initiate gender-affirming hormone therapy in concordance with clinical guideline recommendations
Source: Front Endocrinol (Lausanne). 2024 May 10;15:1086158. doi: 10.3389/fendo.2024.1086158 (PMC11116601; doi:10.3389/fendo.2024.1086158)
Supplement: Supplementary file 1 [file Table_1.docx]

**Supplementary Table 1.** List of gender identity disorder diagnosis codes

| **Code Type** | **Code** | **Description** |
| --- | --- | --- |
| ICD-9 | 302.3 | Transvestic fetishism |
| ICD-9 | 302.51 | Trans-sexualism with asexual history |
| ICD-9 | 302.52 | Trans-sexualism with homosexual history |
| ICD-9 | 302.53 | Trans-sexualism with heterosexual history |
| ICD-9 | 302.6 | Gender identity disorder in children |
| ICD-9 | 302.85 | Gender identity disorder in adolescents or adults |
| ICD-10 | F64.0 | Transsexualism |
| ICD-10 | F64.1 | Dual role transvestism |
| ICD-10 | F64.2 | Gender identity disorder of childhood |
| ICD-10 | F64.8 | Other gender identity disorders |
| ICD-10 | F64.9 | Gender identity disorder, unspecified |
| ICD-10 | F65.1 | Transvestic fetishism |
| ICD-10 | Z87.890 | Personal history of sex reassignment |
