# Supplementary material for: Clinicians in the Veterans Health Administration initiate gender-affirming hormone therapy in concordance with clinical guideline recommendations
Source: Front Endocrinol (Lausanne). 2024 May 10;15:1086158. doi: 10.3389/fendo.2024.1086158 (PMC11116601; doi:10.3389/fendo.2024.1086158)
Supplement: Supplementary file 2 [file Table_2.docx]

**Supplementary Table 2.** List of diagnosis codes for health conditions

| **Health condition** | **Diagnoses code** |
| --- | --- |
| *Alcohol Use Disorder* | |
| ICD-9 | 291, 303, 305.0, 357.5, 425.5, 535.3, 571.0-571.3, 790.3, 980 |
| ICD-10 | F10, G62.1, I42.6, K29.2, K70, T51.0 |
| *Anxiety* |  |
| ICD-9 | 300.00, 300.01 , 300.02, 300.09, 300.10, 300.20, 300.21, 300.22, 300.23, 300.29, 300.3 |
| ICD-10 | F40, F41 |
| *Depression* |  |
| ICD-9 | 296.2, 296.3, 296.82, 300.4, 311 |
| ICD-10 | F32, F33, F43.21, F43.23 |
| *Drug Use Disorder* |  |
| ICD-9 | 304.2, 304.3, 304.4, 304.5, 304.6, 304.7, 304.8, 305.3, 304.9, 305.2, 305.4, 305.5, 305.6, 305.7, 305.9, 292 |
| ICD-10 | F11, F12, F13, F14, F15, F16, F18, F19 |
| *Posttraumatic Stress Disorder* |  |
| ICD-9 | 309.81 |
| ICD-10 | F43.1 |
| *Tobacco Use* |  |
| ICD-9 | 305.1, V15.82 |
| ICD-10 | F17.2, Z71.6, Z72.0, Z87.891 |
| *Atherosclerosis* |  |
| ICD-9 | 429 |
| ICD-10 | I25 |
| *Stroke* |  |
| ICD-9 | 434 |
| ICD-10 | I63 |
| *Diabetes* |  |
| ICD-9 | 249, 250 |
| ICD-10 | E08, E10, E11, E13 |
| *Hyperlipidemia* |  |
| ICD-9 | 272 |
| ICD-10 | E78 |
| *Hypertension* |  |
| ICD-9 | 401, 402 |
| ICD-10 | I10, I11, I12, I13 |
| *Myocardial Infarction* |  |
| ICD-9 | 410 |
| ICD-10 | I21 |
| *Venous Thromboembolism* |  |
| ICD-9 | 415, 451, 452, 453, 459.1 |
| ICD-10 | I80, I81, I82 |
| *Human Immunodeficiency Virus (HIV)* |  |
| ICD-9 | 042, V08 |
| ICD-10 | B20, Z21 |
