# Supplementary material for: Clinicians in the Veterans Health Administration initiate gender-affirming hormone therapy in concordance with clinical guideline recommendations
Source: Front Endocrinol (Lausanne). 2024 May 10;15:1086158. doi: 10.3389/fendo.2024.1086158 (PMC11116601; doi:10.3389/fendo.2024.1086158)
Supplement: Supplementary file 3 [file Table_3.docx]

**Supplementary Table 3a.** Diagnosis codes for identifying social stressors

| **Code Type** | **Code** | **Description** |
| --- | --- | --- |
| *Violence* | | |
| ICD-9 | 995.8x | Adult maltreatment |
| ICD-9 | E960.0 | Unarmed fight or brawl |
| ICD-9 | E960.1 | Rape |
| ICD-9 | E961 | Assault by corrosive or caustic substance |
| ICD-9 | E962 | Assault by poisoning |
| ICD-9 | E963 | Assault by hanging/strangulation |
| ICD-9 | E964 | Assault by submersion |
| ICD-9 | E965 | Assault by firearms |
| ICD-9 | E966 | Assault by cutting/piercing |
| ICD-9 | E967 | Perpetrator of child and adult abuse |
| ICD-9 | E968 | Assault by other or unspecified means |
| ICD-9 | E969 | Late effects of injury purposely inflicted by another person |
| ICD-9 | E970 | Injury due to legal intervention by firearms |
| ICD-9 | V15.4 | History of trauma |
| ICD-9 | V61.11 | Counseling for victim of spousal and partner abuse |
| ICD-9 | V61.21 | Counseling for victim of child abuse |
| ICD-9 | V62.83 | Counseling for perpetrator of physical or sexual abuse |
| ICD-9 | V71.5 | Observation following alleged rape |
| ICD-9 | V71.6 | Observation following other inflicted injury |
| ICD-9 | V71.81 | Abuse and neglect |
| ICD-10 | O9A.3-5 | Abuse complicating pregnancy |
| ICD-10 | T74 | Neglect or abandonment, confirmed |
| ICD-10 | T76 | Neglect or abandonment, suspected |
| ICD-10 | X92 | Assault by drowning and submersion |
| ICD-10 | X93 | Assault by handgun discharge |
| ICD-10 | X94 | Assault by rifle, shotgun and larger firearm discharge |
| ICD-10 | X95 | Assault by other and unspecified firearm and gun discharge |
| ICD-10 | X96 | Assault by explosive material |
| ICD-10 | X97 | Assault by smoke, fire and flames |
| ICD-10 | X98 | Assault by steam, hot vapors and hot objects |
| ICD-10 | X99 | Assault by sharp object |
| ICD-10 | Y00 | Assault by blunt object |
| ICD-10 | Y01 | Assault by pushing from high place |
| ICD-10 | Y02 | Assault by pushing or placing victim in front of moving object |
| ICD-10 | Y03 | Assault by crashing of motor vehicle |
| ICD-10 | Y04 | Assault by bodily force |
| ICD-10 | Y07 | Perpetrator of assault, maltreatment and neglect |
| ICD-10 | Y08 | Assault by other specified means |
| ICD-10 | Y09 | Assault by unspecified means |
| ICD-10 | Y35 | Legal intervention injury |
| ICD-10 | Y36 | Operations of war |
| ICD-10 | Y37 | Military operations |
| ICD-10 | Y38 | Terrorism |
| ICD-10 | Z04.4 | Encounter for examination and observation following alleged rape |
| ICD-10 | Z04.7 | Encounter for examination and observation following alleged physical abuse |
| ICD-10 | Z04.81 | Encounter for examination and observation following forced sexual exploitation |
| ICD-10 | Z65.5 | Exposure to disaster, war and other hostilities |
| ICD-10 | Z69 | Encounter for mental health services for victim and perpetrator of abuse |
| ICD-10 | Z91.4 | Personal history of psychological trauma |
| *Social/Familial Problems* | | |
| ICD-9 | V62.4 | Social maladjustment |
| ICD-10 | Z59.2 | Discord with neighbors, lodgers and landlord |
| ICD-10 | Z59.3 | Problems related to living in residential institution |
| ICD-10 | Z55 | Problems related to education or literacy |
| ICD-10 | Z60 | Problems related to social environment |
| ICD-10 | Z62 | Problems related to upbringing |
| ICD-10 | Z63 | Problems in relationship with spouse or partner |
| *Housing Instability* | | |
| ICD-9 | V60.0 | Lack of housing |
| ICD-9 | V60.1 | Inadequate housing |
| ICD-10 | Z59.0 | Homelessness |
| ICD-10 | Z59.1 | Inadequate housing |
| *Employment or Financial Problems* | | |
| ICD-9 | V60.2 | Inadequate material resources |
| ICD-9 | V62.0 | Unemployment |
| ICD-10 | Z56 | Problems related to employment/unemployment |
| ICD-10 | Z59.4 | Lack of adequate food and safe drinking water |
| ICD-10 | Z59.5 | Extreme poverty |
| ICD-10 | Z59.6 | Low income |
| ICD-10 | Z59.7 | Insufficient social insurance and welfare support |
| ICD-10 | Z59.8 | Other problems related to housing and economic circumstances |
| ICD-10 | Z59.9 | Problem related to housing and economic circumstances, unspecified |
| *Legal Issues* | | |
| ICD-9 | V62.5 | Legal circumstances |
| ICD-10 | Z65.0 | Conviction in civil and criminal proceedings without imprisonment |
| ICD-10 | Z65.1 | Imprisonment and other incarceration |
| ICD-10 | Z65.2 | Problems related to release from prison |
| ICD-10 | Z65.3 | Problems related to other legal circumstances |
| ICD-10 | Z65.4 | Victim of crime and terrorism |
| ICD-10 | Y92.14 | Prison as the place of occurrence of the external cause |
| *Nonspecific Psychosocial Needs* | | |
| ICD-9 | V62.89 | Other psychological or physical stress |
| ICD-10 | Z65.8 | Other specified problems related to psychosocial circumstances |
| ICD-10 | Z65.9 | Problem related to unspecified psychosocial circumstances |

**Supplementary Table 3b: VHA stop codes for identifying social stressors**

| **Code** | **Description** |
| --- | --- |
| *Violence* | |
| 524 | Active duty sexual trauma |
| *Housing Instability* | |
| 504/511 | Grant and per diem |
| 507/522/530 | Department of Housing and Urban Development- Veterans Affairs Supportive Housing |
| 508/528/529 | Health Care for Homeless Veterans |
| 555/556 | Homelessness/job Rehabilitation |
| *Unemployment or Financial Problems* | |
| 208/222/568/574 | Compensated work therapy |
| 555 | Employment services |
| 535/574 | Job rehabilitation |
| *Legal Issues* | |
| 591 | Incarcerated re-entry |
| 592 | Veterans Justice Outreach |

**Supplementary Table 3c: VHA health factors for identifying social stressors**

| **Social Stressor** | **VHA Health Factor** |
| --- | --- |
| *Violence* | Presenting issue—abuse |
| *Social/Familial Problems* | Social support concerns |
| *Housing Instability* | Housing concerns |
|  | Living situation—shelter |
|  | Presenting issue—housing |
| *Unemployment or Financial Problems* | Economic concerns |
|  | Income source—public assistance |
|  | Income source—unemployment benefits |
|  | Presenting issue—financial |
| *Legal Issues* | Presenting issue—legal |
| *Lack of Access to Care/Transportation* | Access to care concerns |
|  | Presenting issue—transportation |
