# Supplementary material for: Clinicians in the Veterans Health Administration initiate gender-affirming hormone therapy in concordance with clinical guideline recommendations
Source: Front Endocrinol (Lausanne). 2024 May 10;15:1086158. doi: 10.3389/fendo.2024.1086158 (PMC11116601; doi:10.3389/fendo.2024.1086158)
Supplement: Supplementary file 4 [file Table_4.docx]

| **Supplementary Table 4.** Guideline Recommendations on Feminizing and Masculinizing GAHT Initiation | |
| --- | --- |
| **VHA Pharmacy Benefits Management Service Guidelines** | |
| **Type of GAHT** | **Guideline** |
| Feminizing | Do not start estrogen if patient has history of venous thromboembolism (VTE) or breast cancer |
| Feminizing | Do not start spironolactone if patient has history of hyperkalemia or acute renal failure |
| Masculinizing | Do not start testosterone therapy if patient is pregnant or has history of breast cancer, prostate cancer, severe renal or hepatic disease, or recent myocardial infraction, stroke, or heart failure |
| Masculinizing | Check hematocrit before starting testosterone |
| Masculinizing | Do not start testosterone if patient has uncontrolled or untreated erythrocytosis |
| Feminizing and Masculinizing | Documentation of gender identity disorder diagnoses required before starting either feminizing or masculinizing GAHT |

GAHT, Gender-affirming hormone therapy; Uncontrolled or untreated erythrocytosis defined as hematocrit > 50%^15^
